# Xenotransplantation in Nephrology: A Narrative Review

**DOI:** 10.3390/jpm16030161

**Published:** 2026-03-14

**Authors:** Alice O’Regan, Johnny Thornton, Elisha Clark, Sam Kant

**Affiliations:** 1 Nephrology Division, Department of Medicine, St. Vincent’s University Hospital, D04 T6F4 Dublin, Ireland; 2 School of Medicine, University College Dublin, D04 T6F4 Dublin, Ireland

**Keywords:** end-stage kidney disease, allotransplantation, xenotransplantation

## Abstract

End-stage kidney disease (ESKD) is a global health challenge, with kidney transplant demand outstripping supply. Allotransplantation remains the gold standard for treatment but organ scarcity leads to prolonged waiting times and high mortality. Xenotransplantation, using genetically modified porcine kidneys, offers a novel and potentially sustainable solution. Genetic engineering and immunosuppression advances have enabled xenotransplantation to transition from a theoretical possibility to feasible solution. This review explores the evolution of xenotransplantation, the scientific advancements in overcoming immunological barriers, and emerging clinical data. Furthermore, we discuss emerging approaches such as central immune tolerance induction, the ongoing risks of cross-species infection, and the ethical and environmental considerations inherent to scaling up porcine organ donation. With the commencement of the first formal clinical trials, progress in the field could transform kidney transplantation, though questions remain regarding long-term outcomes and societal impact.

## 1. Introduction

End-stage kidney disease (ESKD) is a major global health burden, with over 2 million patients dependent on renal replacement therapy (RRT) [1]. Kidney transplantation represents the gold standard of care for ESKD, outperforming dialysis in survival and quality-of-life outcomes [2]. However, current practice places these patients at the centre of a stark paradigm: for one to receive a kidney, another must donate theirs. As a result, the use of allotransplantation is restricted by a persistent mismatch between organ supply and demand, despite ongoing efforts to expand both deceased and living donation. Waiting lists continue to grow, patients die while waiting, and many will never be listed at all.

Xenotransplantation, defined as the grafting or transplantation of living cells, tissues or organs between members of different species, offers a fundamentally different approach. In the context of ESKD, current efforts focus on utilising genetically modified porcine kidneys as an additional, renewable organ source. Advances in genetic engineering and immunosuppression have moved xenotransplantation from a hypothetical concept towards early clinical application. This emerging field is poised to redefine kidney transplantation, transforming it from a donor-dependent and opportunistic procedure into a planned intervention supported by an on-demand organ supply, with profound implications for survival and quality of life worldwide.

In this review, we examine the history of xenotransplantation and the advances that have been made over time. Secondly, we explore the science underpinning xenotransplantation and discuss emerging trials. Finally, we explore future directions and areas of promise in xenotransplantation.

## 2. History of Xenotransplantation

Human fascination with blending elements of different animals predates modern science, with hybrid creatures populating ancient mythology. This intellectual curiosity evolved into experimental practice in the late 17th century when French physician Denys transfused the blood of a lamb into a 15-year-old boy [3]. Dutch surgeon van Meekeren subsequently performed one of the first documented xenografts, using a segment of canine skull to fix a cranial defect in a Russian soldier [4]. However, early xenotransplantation attempts were sporadic, unsuccessful and provoked public discomfort, causing enthusiasm to wane.

It was not until 1902 that surgical pioneer Alexis Carrel successfully performed the first heterotopic kidney transplant in a canine, inserting the animal’s own kidney into its neck. He observed the hostile host responses to the xenografts, attributing this to intrinsic biological factors rather than surgical failure [5]. Building on this work, the first documented attempt at kidney xenotransplantation is attributed to Princeteau, a French surgeon. In 1905, he transplanted slices of rabbit kidney into a child with chronic kidney disease, who died 16 days later [6]. A year later, Jaboulay performed the first vascularised kidney xenotransplants, from a pig and a goat, into patients with irreversible kidney failure. Initially, the grafts were seen to produce urine but were removed on the third day after they stopped functioning [7].

A major milestone occurred in 1954, when Joseph Murray performed the first successful human organ transplant: a kidney transplant between identical twins, enabled by advances in vascular surgery, anaesthesia and perioperative care [8]. Between 1963 and 1964, Reemtsma transplanted kidneys from chimpanzees into thirteen patients; most died within weeks, yet one patient survived for 9 months, even returning to work, before dying from a presumed electrolyte disturbance [9]. Transplant pioneer Thomas Starzl highlighted the significance of these experiments in his seminal book *Experience in Renal Transplantation* [10]. He recognised that problems with the procurement of human kidneys would greatly limit widespread allotransplantation and that more effective immunosuppression was required to enable the use of animal donors for xenotransplantation [10]. Two years later, in 1966, Starzl performed the first chimpanzee-to-human liver xenotransplantation and went on to lead several further cases over the ensuing eight years. Despite these efforts, graft function was short-lived, with none surviving beyond two weeks [11,12]. In the wake of this, xenotransplantation receded to the margins as nothing more than a scientific curiosity, whereas allotransplantation entered the spotlight as a primary treatment for organ failure.

**Figure 1:**
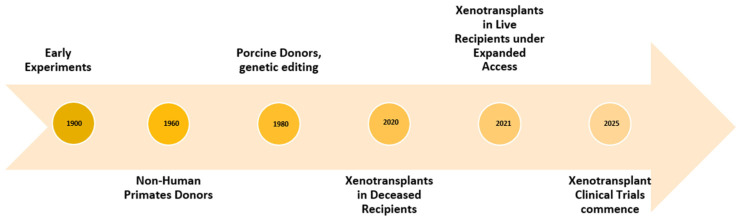
Timeline of the progression of xenotransplantation in the 20th century.

The modern era of xenotransplantation began in the late 20th century (see Figure 1), revived by the discovery of modern immunosuppressive drugs such as calcineurin inhibitors (CNIs) and furthered by genetic manipulation. In a landmark study, Anand et al. demonstrated that xenotransplantation of a humanised porcine kidney graft into a non-human primate (NHP), combined with an appropriate immunosuppressive regimen, supported long-term survival of 758 days [13].

## 3. Why Is Xenotransplantation Needed?

Allotransplant remains the preferred option for RRT when compared with dialysis. Studies performed by Zhang et al. demonstrated a survival advantage of 13.8 years in transplant patients, with reduced healthcare costs over the first three years post-transplant [14,15]. Importantly, recipients have a better quality of life with a lower risk of cardiovascular events, despite increases in age and comorbidity over time [2].

According to the Global Observatory on Donation and Transplantation (GODT), in 2024, there were 110,467 kidney transplants performed worldwide, making it the most frequently undertaken solid organ transplant procedure. Despite this, transplantation remains available to only a fraction of those who might benefit, and global figures likely underestimate true need given barriers to listing and access to transplant programmes. In the U.S. alone, OPTN registry data show that as of January 2026, there were 94,216 ESKD patients on the national transplant waitlist, yet only 27,574 transplants (living and deceased donor combined) were performed in 2025 [16,17]. This represents 3.4 patients waiting for each kidney transplant performed, clearly illustrating that demand exceeds supply. Thousands die each year while awaiting kidney transplantation, even in large transplant programmes, where waiting times can extend to several years. A significant proportion of patients on the waitlist become too unwell to proceed or die with complications of their ESKD, with over 7000 deaths on solid organ waiting lists reported worldwide in 2023 [16]. Many more patients will never make the waitlist, due to comorbidities, limited availability of transplant centres and geographical inequity.

The central problem in modern allotransplantation is the scarcity and unpredictability of organ supply. In response, governments have developed living donor transplantation, paired exchange programmes, structured national donor-coordination programmes like UNOS and the presumed-consent (“opt-out”) policies. However, demand is unlikely to be met through deceased and living donation alone. The incidence of suitable donor death, variable organ quality and challenges with consent processes all limit supply.

Xenotransplantation has risen to prominence as a potential strategy to bridge the gap. By generating transplantable kidneys from genetically modified pigs, xenotransplantation proposes a scalable and predictable source of organs independent of human donation. In principle, this could shorten transplant waitlists, reduce dialysis numbers and extend access to transplantation to those unlikely to ever receive a kidney.

As xenotransplantation aims to transition from an experimental intervention to a sucessful treatment, careful appraisal of its scientific foundations, the emerging clinical data and the ethical and regulatory implications is critical.

## 4. Immunobiology of Xenotransplantation

Recent advances in xenotransplantation hinge on deciphering and modulating the complex immunological barriers that threaten graft survival. The interplay between the recipient’s immune system and the porcine organ gives rise to a series of immunological responses, involving the innate and adaptive immune system. Various genetic and pharmacological methods have been developed to overcome these barriers.

Hyperacute rejection, the first significant hurdle, is mediated by preformed natural antibodies (primarily anti-Gal) that recognise carbohydrate xenoantigens such as galactose-α-1,3-galactose (Gal) on porcine vascular endothelium. Binding of these antibodies triggers complement activation and vascular injury, often resulting in graft thrombosis and loss within minutes to hours post-transplant [18,19,20]. Overcoming hyperacute rejection in large part has been achieved through genetic engineering of pigs lacking key xenoantigens, such as α1,3-galactosyltransferase knockout (GTKO) animals, and expressing human complement regulatory proteins—DAF/CD55, MCP/CD46 and CD59—which dampen complement-mediated injury [20].

Even with reduced antibody and complement reactivity, robust innate immune responses remain a major threat. Macrophages, monocytes, neutrophils, and especially natural killer (NK) cells play significant roles in rejection. Macrophages and NK cells attack the xenograft directly or mediate damage via antibody-dependent cellular cytotoxicity (ADCC), facilitated by the binding of recipient immunoglobulins deposited on the graft endothelium. CD47, a self-recognition molecule, is a crucial modulator. Porcine CD47 is poorly recognised by human signal regulatory protein α (SIRPα), failing to deliver inhibitory signals to recipient macrophages. Transgenic expression of human CD47 in donor pigs has emerged as a way to suppress macrophage-mediated phagocytosis. Neutrophils infiltrate xenografts early and extrude neutrophil extracellular traps (NETs), which inflict tissue damage. Macrophages, responding to tissue injury signals (DAMPs), further amplify local inflammation by cytokine production, exacerbating the rejection cascade [18,21,22,23].

Adaptive immunity is the final, long-term barrier to xenograft acceptance. Both T- and B-cells participate in direct and indirect recognition of porcine antigens. In the direct pathway, human T-cells recognise porcine MHC (swine leukocyte antigen, SLA) molecules, leading to T-cell activation, clonal expansion, and cytotoxicity. The indirect pathway involves recipient antigen-presenting cells processing porcine peptides and presenting them to helper T-cells, which activate B-cells and stimulate production of xenoreactive antibodies [19,20,21,22,23,24]. Recent studies have demonstrated that genetically modified kidney xenografts remain vulnerable to both T-cell-mediated rejection (TCMR) and antibody-mediated rejection (ABMR) in humans and NHPs. Some patients have developed early TCMR reversed by T-cell-depleting antibodies, yet long-term control of humoral responses remains challenging [22,25].

An emerging area centred on central tolerance is under study involving the insertion of thymic tissue under the renal capsule of porcine kidneys prior to transplantation (thymokidneys). These aim to “educate” the recipient’s immune system to accept porcine antigens as self, blunting both T- and B-cell responses [26]. This has shown some promise, with no evidence of hyperacute rejection seen in these cases [27,28].

Immunosuppressive regimens have evolved alongside genetic engineering. Protocols use more intensive induction and costimulation-based maintenance regimens than standard allotransplantation, reflecting the higher innate and adaptive immune barriers to a porcine graft (See Table 1) [29,30,31,32].

In contemporary pig-to-NHP and early pig-to-human kidney models, induction is typically “multi-hit” to target innate, humoral, and cellular responses simultaneously. These regimens frequently combine: lymphocyte depletion (rabbit antithymocyte globulin) to reduce T-cell burden pre-implantation and attenuate early cellular rejection; B- and plasma-cell targeting with rituximab, aiming to reduce pre-existing and de novo anti-pig antibodies; complement blockade (eculizumab) or related C5 inhibitors to mitigate residual antibody-mediated injury and features of delayed xenograft injury analogous to ABMR; and cytokine and innate immune modulation (e.g., TNF-α blockade with etanercept) to dampen early inflammatory cascades triggered by cross-species recognition and ischemia–reperfusion [32,33]. These are usually combined with early initiation of CD40/CD154 costimulation blockade rather than relying solely on depleting antibodies plus CNIs [29,33].

Maintenance immunosuppression in xenotransplantation centres on sustained costimulation blockade, most commonly of the CD40–CD154 axis, layered onto conventional agents. Long-term administration of anti-CD154 (CD40L) or anti-CD40 monoclonal antibodies acts as the principal T-cell inhibitor, with meta-analytic data in NHP kidney transplantation showing superior rejection-free survival and more durable tolerance-like states with anti-CD154 compared to anti-CD40 [27]. Concomitant use of mTOR inhibition has been studied, which not only suppresses T-cell proliferation but also expands regulatory T-cells and helps control proliferative pig graft changes. However, this is at the cost of gastrointestinal toxicity and a narrow therapeutic window, predisposing to humoral rejection when under-dosed. Some regimens focus on adjunct low-dose CNIs and antimetabolites, providing additional suppression of effector T-cell and B-cell responses while costimulation blockade remains the dominant signal-2 inhibition [32]. Overall, xenotransplant maintenance tends to prioritise biologic costimulation blockade plus mTOR-based strategies, often with continued complement- or B-cell-directed therapy in higher-risk settings, rather than relying solely on CNI-based triple therapy. Conventional CNI-based triple therapy remains the backbone of human allotransplantation, with biologic induction tailored mainly to immunological risk rather than to a fundamentally different cross-species immune response [29,32].

## 5. Real-World Data

The concept of using closely related species to address the shortage of organs is not novel and was trialled as early as the 1960s. Significant advances in gene editing over the past 20 years have made the xenotransplant a credible solution to address the current organ shortage that exists globally.

Three centres have carried out xenotransplants under varying conditions over the past five years: the University of Alabama, Massachusetts General Hospital and NYU Langone Health [23,27,28,34,35,36]. Initial trials have involved decedent recipients, which have allowed researchers to conduct in vivo assessments of the feasibility of porcine xenotransplantation. The University of Alabama has invested heavily in xenotransplantation with the development of a specific programme in 2015 involving a designated animal facility, xenotransplant centre and a workforce with extensive experience in the field. They published two cases of xenotransplants in decedent recipients in 2022 using porcine kidneys with 10 gene edits. Recipients had previously undergone bilateral nephrectomies to establish anuria, with the index case immediately producing urine from the graft. Despite good urine output, there was no corresponding fall in serum creatinine. Serial biopsies showed thrombotic microangiopathic (TMA) changes and subsequently tubular injury as the study progressed. Standard induction immunosuppression comprised ATG and methylprednisolone, followed by a CNI-based maintenance regimen. A further study was conducted under similar conditions—a key difference being that the deceased recipient received eculizumab. Research suggests it may assist in preventing TMA changes in the porcine graft. In contrast to the previous study, serum creatinine decreased within the first 24 h and remained within the normal range for the duration of the study (7 days). Serial biopsies of the xenografts showed normal histology [32,33,36].

Montgomery et al. at NYU Langone have also published findings on two cases of porcine kidney xenotransplants in decedent recipients. Both xenografts produced urine within minutes of reperfusion with serum creatinine steadily decreasing over the course of the study. Of note, the decedent did not undergo bilateral nephrectomies prior to xenotransplant and was not anuric, indicating the native kidneys may have contributed to creatinine reduction. Electrolyte abnormalities were noted, namely hypernatremia (thought to be attributed to the development of arginine vasopressin deficiency in the brain-dead individual) and hypokalemia (a finding that can be present post-transplant involving human kidneys). Serial biopsies showed no evidence of ABMR or TCMR. The study terminated as planned at 54 h. Despite the short study length, both xenografts showed good function with no evidence of hyperacute rejection [23]. A key difference between centres was the immunosuppressive regimens used, with NYU focused on the theory of central tolerance and utilisation of the “thymokidney”. Previous research has shown that this is a means to reduce the risk of T-cell-mediated rejection, and biopsies from these cases demonstrated no evidence of T-cell-mediated injury [34].

Building on the research in deceased recipients, two cases of kidney xenotransplant in living recipients were carried out at NYU Langone. Both cases were approved by the U.S. Food and Drug Administration (FDA) via their expanded access programme. The first recipient (54) with a background of cardiac and renal failure received an LVAD followed by a porcine kidney transplant. They died 47 days post-transplantation. The latter (53) was a patient with a history of ESKD secondary to previous nephrectomy and hypertension. The graft functioned for a total of 130 days prior to explantation following an episode of acute rejection [37,38].

Kawai et al. at Massachusetts General Hospital published a further case of a xenotransplant in a living recipient. The male recipient (62) had ESKD with a calculated risk of 76% of being excluded from receiving a transplant due to poor health or dying within the next 5 years. A porcine kidney with a total of 69 gene edits was utilised. A distinctive immunosuppressive regimen to those previously described was used, which included ATG, rituximab, tegoprubart (anti-CD154 monoclonal antibody) and ravulizumab (anti-C5 antibody). Maintenance immunosuppression included tacrolimus, mycophenolic acid and prednisolone. The post-operative course was complicated by an episode of TCMR identified on renal biopsy on day 8 following rising serum creatinine. Immunosuppression was augmented, with the recipient receiving a total of three doses of pulsed methylprednisolone, tocilizumab, and an increase in his tacrolimus and mycophenolic acid. Pegcetacoplan, a targeted C3 and C3b inhibitor, was also introduced due to evidence of C3 deposition on the biopsy. A further biopsy revealed resolution of the TCMR. The recipient was discharged on day 18 post-operatively. The recipient died on day 51 post-transplantation—concluded to be due to sudden cardiac death secondary to a cardiac arrhythmia on the background of severe ischaemic cardiomyopathy [35]. A further xenotransplant involving a live recipient has been performed, although the findings await publication [39].

Building on the progress to date, NYU Langone in partnership with United Therapeutics has recently received approval to commence EXPAND, a single-arm, phaseless clinical trial aiming to evaluate the efficacy and safety of porcine xenotransplants for ESKD patients. An initial cohort of six patients have been recruited who are 55–70 years old, have been on dialysis for at least 6 months and have been deemed ineligible for an allograft for medical reasons or because they are more likely to die while awaiting an allograft [40,41]. A summary of recent xenontransplants is detailed in Table 2.

## 6. Future Directions

The approval and subsequent commencement of xenotransplant clinical trials signals the momentum that xenotransplantation is gathering as a viable solution to address the significant organ shortage. Even if xenotransplant immunobiology can be successfully navigated, multiple barriers still exist that may hinder the adoption of xenotransplant programmes worldwide.

### 6.1. Infectious Disease Risks and Mitigation in Xenotransplantation

Mitigating the risk of infectious disease remains a challenge and will require a novel approach to both recipients and donors. Concern remains regarding porcine pathogens and porcine endogenous retrovirus (PERV) despite success using CRISPR technology to inactivate the PERV within the pig genome [42]. In trials conducted to date, all donors underwent extensive screening for possible porcine pathogens prior to donation using a variety of methods. A standardised protocol of which pathogens to test for and how and when to test for them does not currently exist. Different methods have been explored, with tools such as metagenomics showing promise in the detection of new or unusual pathogens. Viruses identified can be considered in distinct categories, pathogenic to pigs and pathogenic to pigs and humans, causing zoonotic infections in humans for which immunosuppressed individuals would be at particular risk. To date, a comprehensive list does not exist. Pathogens identified to be of concern include porcine cytomegalovirus causing graft rejection, PERVs and porcine cornoviruses [42]. Alongside genetic engineering, controlled breeding environments will be key to minimising pathogen exposure, particularly those established to build a biosecure herd.

As with allotransplantation, a through pre-transplant infectious disease assessment will be essential for blood-borne viruses and latent infections such as TB. Post-transplantation, the FDA (CBER) requires all xenotransplant recipients to consent to lifelong infectious disease monitoring, indicating the need for a robust standardised post-transplant surveillance programme. Consideration needs to be given to retaining serological samples of both recipients and close contacts to allow for the monitoring of either serological or molecular evidence of infection in the future. This will have implications both for the consent of potential recipients but also for how transplant programmes will structure the long-term follow-up of these patients. Xenotransplantation raises population-level concerns about the potential of a novel zoonotic infection arising which could spread beyond that of the individual recipient [43,44,45]. This is distinctive to allotransplantation, reflecting the unique public health dimension associated with xenotransplantation.

### 6.2. Ethical Concerns

Xenotransplantation raises complex ethical issues, particularly surrounding individual autonomy and equity. The risk of zoonotic transmission is difficult to predict, resulting in the need for long-term surveillance of individuals and their contacts with potential privacy implications. It is not yet clear as to who will retain this data, how long for and if the recipient is eligible to withdraw from surveillance in the future. It is imperative that patients are fully informed of their responsibilities at the point of consent. Animal welfare must also be considered as raising animals for use in xenotransplantation differs from animals raised for food production. Porcine donors are genetically modified and exist in isolation in a biosecure unit in complete contrast to their natural environment, likely causing pain and suffering. Animal rights groups have voiced their unease regarding xenotransplantation on the basis that pigs are sentient and intelligent beings with the capacity to experience pain and suffering.

The hope is that xenotransplantation will offer an alternative to allotransplantation, although ensuring equitable access will likely prove challenging. As with all novel treatments, significant financial investment is required, resulting in the benefits being limited to more privileged groups initially. Despite ongoing research and advancement, allografts may prove superior in terms of graft function and safety. Allografts may be a patient’s preference, or xenografts may be unacceptable in line with a patient’s belief system, which will raise concerns about access and equity [46,47].

## 7. Conclusions

Xenotransplantation continues to evolve and currently represents a promising strategy to address the global organ shortage. Advances in gene editing and immunomodulatory therapies have provided progress, although long-term graft survival has yet to be proven. Questions regarding risk of cross-species infection, control of xeno-immune response and ethical concerns remain. Further research and careful oversight are required to support the development of xenotransplantation into a viable solution for patients with ESKD.

## Figures and Tables

**Table 1:** Summary of the contrasting immunobiology in xenotransplantation and allotransplantation.

	Xenotransplantation	Allotransplantation
Immune Barrier Target	Cross-species innate, humoral and cellular xenogenic responses	Primary allo-reactive T-cell antibody responses
Induction Agents	Multi-agent: ATG, rituximab, complement and TNF-alpha blockage, plus early costimulation blockade	Usually single biologic (basiliximab or ATG) tailored to immune risk
Maintenance Agents	CD40/CD154 costimulation blockade, often with mTOR inhibition as a key scaffold	CNI-based triple therapy (tacrolimus, mycophenolate, and steroids) as standard
Tolerance and Durability	Anti-CD154 regimens in NHPs can induce prolonged “nonresponse” after withdrawal, but long-term safety and thrombosis risk in humans remain under study	Operational tolerance rare; long-term CNI exposure limited by nephrotoxicity and metabolic effects

**Table 2:** Summary table of past and current research in renal xenotransplantation.

Year	Research Group	Recipient	Donor	Immunosuppression	Duration	Outcome
2021	University of Alabama	Deceased	10 Gene-Edit Pigs	ATG 6 mg/kg Rituximab (Anti-CD20) Methylprednisolone Tacrolimus	74 h	Immediate urine output, no fall in serum creatinine Histological evidence of TMA and tubular injury
2023	University of Alabama	Deceased	10 Gene-Edit Pigs	ATG Rituximab (Anti-CD20) Eculizumab (Anti-C5 inhibitor) Methylprednisolone Tacrolimus Mycophenolate Mofetil	7 days	Immediate graft function with urine output and falling serum creatinine Normal Histology No evidence TMA [32,33]
2022	NYU Langone	Deceased	GTKO pig kidney + donor-specific thymic tissue	Thmyokidney Methylprednisolone Mycophenolate Mofetil	54 h	Immediate graft function with good urine output and falling serum creatinine No evidence of hyperacute rejection, ABMR or TCMR [24]
2023	NYU Langone	Deceased	Single gene-edited porcine donor	Unpublished, press release	47 days	
2024	NYU Langone	Living	U Kidney, 10 gene-edits	Unpublished, press release	130 days	
2025	Massachusetts General Hospital	Living	Yucatan miniature pig 69 genomic edits (eGenesis)	ATG Rituximab (Anti-CD20) Tegoprubart (Anti-CD154 FC modified monoclonal antibody) Ravlizumab (Anti-C5 antibody) Tacrolimus Mycophenolic acid Prednisolone	52 days	Immediate graft function Episode of TCMR, resolved with increased immunosuppression No evidence of ABMR or TMA changes Recipient death from cardiac cause [35,39]
2025	Massachusetts General Hospital	Living	Gene edited pig (eGenesis)	Unpublished, press release	271 days	Unpublished

## Data Availability

No new data were created or analysed in this study. Data sharing is not applicable to this article.
